# Three methods of estimating age‐at‐death using three different regions on the os coxa

**DOI:** 10.1111/1556-4029.70219

**Published:** 2025-11-10

**Authors:** Sthembiso S. Mkhonza, Ntombifuthi P. Ngubane, Okikioluwa S. Aladeyelu, Carmen O. Rennie

**Affiliations:** ^1^ Discipline of Clinical Anatomy, College of Health Sciences University of KwaZulu‐Natal Durban South Africa; ^2^ Faculty of Medicine and Health Sciences Walter Sisulu University Mthatha South Africa

**Keywords:** acetabulum, age‐at‐death estimation, auricular surface, forensic anthropology, os coxa, population affinity, pubic symphyseal surface

## Abstract

Understanding population diversity is necessary for the development of forensic anthropology methods that address population affinity. The commonly used age‐at‐death estimation methods for adult skeletal remains are based on American data sets, which include individuals with both European and African population affinities. Evaluating these methods on different skeletal collections worldwide is hampered by our incomplete understanding of population diversity. Hence, this study evaluated three methods of estimating age‐at‐death using the pubic symphyseal surface, auricular surface, and acetabulum regions on the os coxa. A total sample size of 143 os coxae from Black South African and White South African population groups was examined in KwaZulu‐Natal (KZN). The accuracy (chi‐squared test of independence), bias, absolute error (mean absolute error, Wilcoxon signed‐rank test), interobserver, and intraobserver correlation coefficients (Cronbach's alpha test) were recorded for each method. The auricular surface method scored the highest accuracy; however, the reliability of this method is still questionable, as it also scored the highest absolute error and bias among the three methods on both population groups. Compared to the pubic symphyseal surface and auricular surface methods, the acetabulum method showed promise, as it maintained lower bias and absolute error and was consistent when re‐evaluated in both populations. Laterality was insignificant for any of the three methods. Sex was insignificant for the acetabulum and auricular surface methods, but significant for the pubic symphyseal surface method in the White South African samples and insignificant in Black South African samples. These results demonstrated the need for creating population‐specific standards, including precisely defined age ranges and reference samples catered to the diverse South African populations.

**Highlights**

- This study examines three age‐at‐death estimation methods using os coxa on a large sample of South Africans.
- The pubic symphysis, auricular surface, and acetabulum methods were evaluated for accuracy and reliability.
- The work here underscores the need for population‐specific standards for the South African population.

## 1 INTRODUCTION

Establishing a person's identity is essential from a medical and legal perspective, especially in cases of murder or mass killings, where the bodies are either terribly mutilated or have already started to decay [1]. Age and sex are the most important identifying factors that eliminate more than half of the challenges when creating an anthropological profile [2]. There are documented methods used for estimating age‐at‐death, and they are based on the morphological degenerative changes in the teeth and bones, with most previous studies from forensic anthropology focusing on the skull, thorax, and pelvis skeletal regions [3]. The pelvis is a commonly used bone in the forensic community, as it gradually varies with age, with certain morphological changes beginning early and continuing into later life [4]. The pelvis is made up of two os coxae or hip bones, and each os coxa is formed by the ischium (curved anterior protuberance), pubis (curved inferior protuberance), and ilium (flat superior protuberance) [5]. The pubis, ilium, and ischium bones are all connected in the acetabulum area by cartilage at birth; subsequently, between the ages of 16 and 18, they fuse to create a single bone [5]. The os coxa is a good landmark for estimating age‐at‐death because it has three age indicators, where bony changes correlate with the advancing age: viz. the pubic symphyseal surface, auricular surface, and the acetabulum [6].

The complex interaction between genes, culture, and environment determines the rate and degree of change, contributing to the unique life history of everyone [7]. Knowledge of whether a given approach is accurate (correct), precise (refined), and repeatable when applied to unidentified individuals outside of the initial reference sample is essential for the effectiveness of identification methods. Several age‐at‐death estimation methods have been created with reference samples from North America and Europe [8, 9, 10]. Todd [11] first documented American standards for utilizing pubic symphyseal surface morphology to estimate age‐at‐death from skeletal remains. These standards were for White males in America before they were developed to include White females and Black males and females. Since the documentation of Todd's [11] work, most researchers have evaluated the applicability and accuracy of his approach for various populations [8]. Katz and Suchey [12] listed the following flaws in the original Todd method: it overestimated the age‐at‐death for most individuals, especially those under 40 years of age; it ignored individual variation; and it aged older individuals with 65+ years inaccurately [12]. Brooks and Suchey [13] evaluated age estimates by two methods requiring os pubis examination. This approach involved the analysis of the degradative morphological alterations in the pubic symphyseal surface. There were six stages to these morphological characteristics that Brooks and Suchey postulated. Brooks and Suchey [13] found that there are physical distinctions between the sexes but concluded that a single set of descriptions can be applied to both sexes because the focus is on important age changes observed in both male and female pubic bones [13].

Lovejoy et al. [14] provided a method that examined age changes in the auricular surface of the ilium. This method consisted of eight phases, each of which was divided into age ranges that corresponded with morphological descriptions [14]. The Lovejoy method did not perform well when estimating age for older individuals of 65+ years. It was also noted that this method tended to overestimate the age of individuals from 18 to 39 years and underestimate the age of older individuals of 65+ years [14]. The auricular aging method holds great potential as Buckberry and Chamberlain [15] sought to enhance the precision and ease of use of the Lovejoy [14] approach. Osborne et al. [16] discovered that there is no difference in accuracy when reducing Lovejoy's eight‐phase approach to six stages, of which the features included were transverse organization, surface texture, microporosity, macroporosity, and changes in morphology of the apex and retro‐auricular area that correspond with specific age ranges and phases [16].

The first investigation on the acetabulum as an age marker in the adult skeleton was conducted by Rouge‐Maillart et al. [17]. They postulated that the acetabulum, the third pelvic joint, was equally relevant in estimating age, citing the usefulness of the auricular surface and pubic symphysis. The acetabulum has age‐related degenerative changes, just like the pubic symphysis and the auricular surface, but it is also resistant to postdepositional degradation [17]. Later, Rissech [4] refined the acetabulum methodology using the Bayesian technique and processed data using a novel statistical tool (IDADE2), which has been previously reported to be more appropriate when analyzing data than linear regression with less bias, higher accuracy, and narrower 95% confidence intervals [18, 19]. Rissech's [4] method showed a strong correlation between observable variables and age, less bias, as well as minimal intra‐ and interobserver error because of the standardized numerical scoring procedure [4]. The method's shortcomings include its novelty and relative lack of testing, as well as the fact that its interobserver reliability and sensitivity to sex and ancestry variations have not yet been determined. It is uncertain if the procedure can be applied to females because it was created using a skeleton sample that was (exclusively) male [4]. Furthermore, it has been suggested that the approach could be susceptible to variations in the population; also, there have been reports of averaging among the extremely young, 15–20 years old, at death [20]. The use of acetabulum alterations as skeletal age markers has been questioned since the hip is a joint that experiences changes because of possibly nonage‐related stresses such as differential mechanical loading, osteoarthritis, and dysplasia [17].

Rissech [17] outlined seven age changes in the acetabulum bone and concluded that acetabular observations allow for precise age‐at‐death estimations for individuals over 40 years of age [17]. The acetabulum approach was re‐evaluated by Calce and Rogers [21], who tested the seven acetabular criteria documented by Rissech [4] in multiple phases. To streamline the acetabulum approach, they first reduced the number of variables from seven to three, along with the descriptions for both males and females' os coxae. Young adults, middle adults, and old adults were the three age stages that were distinguished by the three observable features (apical growth, osteophyte development of the acetabular rim, and acetabular groove) [22]. The auricular surface of the ilium and the acetabulum are two areas of the os coxa that have been reported in earlier research to be resilient to postdepositional events, including weathering and breakage [23].

Adult age estimation has been modified significantly throughout the history of forensic anthropology. The common issue with the abovementioned traditional age‐at‐death estimation methods was population specificity, age mimicry (i.e., when the target sample reflects the age distribution of the reference sample used to create the age indicators), bias, and accuracy [24]. Hence, most studies evaluated and improved the existing age‐at‐death estimation methods and discovered new age markers for various populations [9]. Currently, skeletal remains are mostly evaluated by South African forensic laboratories using four morphological methods, viz. the cranial suture closure [14, 25], the symphyseal face of the pubis [13], the auricular surface of the ilium [14], and the sternal end of the fourth rib [26]. However, the transition analysis tool ADBOU 2.1 software combines the last three methodologies [27, 28]. Due to how it produces a maximum likelihood age estimate based on the accuracy and reliability of the age indicators, transition analysis has become widely accepted as a solution to the problem of the reference sample's age mimicry [8].

Numerous studies tested transition analysis, Bayesian statistics, and multifactorial vs. single approaches for assessing the pubic symphyseal surface. The Bayesian strategy outperformed the Suchey‐Brooks single method, according to Godde and Hens [24]. It was also indicated that the age estimates for the target sample that were produced were influenced by the population from which the transition analysis parameters were derived. This means that while the pubic symphysis undergoes age‐progressive morphological changes, various populations experience these changes on different timelines and along distinct trajectories, which might affect the final age‐at‐death estimation [24]. The validity of the Suchey‐Brooks and Buckberry‐Chamberlain approaches was investigated by Villa [29] using 3D representations from computed tomography (CT) and laser images, and concluded that although modern imaging technology can improve these methods, direct viewing of dry bones now yields the most accurate results [29]. The necessity of enhancing the os coxa age‐at‐death estimation techniques to critically assess substitute strategies that make use of the pubic symphyseal surface and auricular surface has also been emphasized [25]. Hence, the purpose of this study was to evaluate the three age‐at‐death estimation methods of Suchey‐Brooks pubic symphyseal surface, Osborne auricular surface, and Calce acetabulum region and document their performance when utilized in Black South African and White South African population groups in KZN.

## 2 MATERIALS AND METHODS

### 2.1 Sample

A total sample of 143 os coxae of Black South Africans (*n* = 92) and White South Africans (*n* = 51) in KZN province was examined in this study. The sample size was separated into sex and laterality (Table 1) and further distributed into age categories (Table 2). The sample was selected from the collection of bones at the University of KwaZulu‐Natal (UKZN) Department of Clinical Anatomy (Nelson R Mandela School of Medicine and Westville campus) and the Durban University of Technology (DUT), Department of Basic Medical Sciences (Steve Biko campus). Ethical clearance was obtained from the Biomedical Research Ethics Committee (BREC) of the UKZN (ethical clearance number: BREC/00006368/2023) and a gatekeeper's permission letter from DUT.

**TABLE 1:** The sample size separated into sex and laterality.

	Males	Females	Left	Right
Black South Africans	58	34	48	44
White South Africans	24	27	30	21
Total	82	61	78	65

**TABLE 2:** The sample size separated into age distribution.

Age group	Black South Africans	White South Africans
18–20	4	–
21–30	10	1
31–40	23	3
41–50	22	3
51–60	23	7
61–70	8	3
71–80	2	15
81–90	–	13
91–100	–	6
Total	92	51

### 2.2 Inclusion/exclusion criteria

The right and left os coxae of individuals, ranging from 18 to 100 years of age, which had adequate historical documentation, were included in this study. Os coxae that had deterioration in any of the three regions of interest that would have rendered some aspects invisible were thus excluded from this study.

### 2.3 Methodology

To avoid bias of the results, mimic the chronological ages of the sample, A blind test was conducted to examine the three age‐at‐death estimation methods where the chronological age‐at‐death, sex, and population group information of each os coxa were made unknown until the end of data collection: viz. the pubic symphyseal surface by Brooks and Suchey [13], auricular surface by Osborne et al. [16], and acetabulum by Calce [22]. These methods were evaluated separately at different times. Each region of interest on the os coxa was carefully examined using one method at a time until all samples were completed before the examination of another method commenced, as the first 2 weeks of data collection were only assigned for the pubic symphyseal surface method until all the samples were completed. Age was then estimated by categorizing each os coxa into different age groups using age ranges corresponding to each method created by Brooks and Suchey [13], Osborne et al. [16], and Calce [22]. These age ranges were separated into different aging phases of the os coxa using descriptions and pictures matching each phase. Although sex differences were deemed insignificant for auricular surface and acetabulum methods by Buckberry and Chamberlain [15] and Rissech and Malgosa [30], the pubic symphyseal surface method had distinct age ranges between males and females [13] (Table 3).

**TABLE 3:** Different age ranges for different age‐at‐death estimation methods, Suchey‐Brooks, Osborne, and Calce.

Phase	Suchey‐Brooks (pubic symphyseal surface) method		Osborne (auricular surface) method	Calce (acetabulum) method
	*Male and female age ranges (years)*		*Both sex age ranges (years)*	*Both sex age ranges (years)*
1	15–23	15–24	≤27	Young adults
2	19–34	19–40	≤46	(17–39)
3	21–46	21–53	≤69	Middle adults
4	23–57	26–70	20–75	(40–64)
5	27–66	25–83	24–82	
6	34–86	42–87	29–89	Old adults (65+)

Using the magnifying glass and adequate lighting, age estimates were assigned by applying the Suchey‐Brooks [13] pubic symphyseal surface phases, along with their descriptions, and by referencing age ranges and pictures. Osborne's approach phase definitions were followed in assigning age estimates to the auricular surfaces [16]. Three acetabular stages—young 17–39 years, middle 40–64 years, and old adult 65+ years—were scored according to descriptions given by Calce [22]. The final estimate referred to the oldest variable when the three indications showed varying ages, as Hartnett [31] advised for the pubic symphyseal surface. The sample was classified as being in the old adult phase; for instance, if the rim appeared young and smooth but the apex appeared old, that is, rough with bone osteophytes accumulation.

### 2.4 Statistical analysis

Three approaches were used to assess the effectiveness of the three aging methods. First, the accuracy was scored. Accuracy was defined as the number of individuals whose known ages fell within the ranges established by each method. Even when the known age fell into the range of multiple phases, it was still considered accurate if it fitted within the age range of that phase and vice versa for inaccuracy [32]. Second, the mean absolute error (MAE) and Wilcoxon signed‐rank test were conducted to compute the bias and absolute error for each method. Whether the estimated age is overestimated or underestimated, bias is the statistical measure that shows the direction of the misclassification error. Positive bias is present if the estimated age exceeds the chronological age. The bias is negative if the estimated age is lower than the chronological age. The average difference between the estimated age and the chronological age using each method was used to compute the bias, which was expressed as $\frac{\sum \;\left(\text{estimated}\;\mathrm{age}-\text{chronological}\;\mathrm{age}\right)}{n}$ according to [2, 6, 33].

The absolute error is a statistical metric used to assess the extent of a method's misclassification error. The average absolute difference between the estimated age and the chronological age for each method $\frac{\sum \;|\text{estimated}\;\mathrm{age}-\text{chronological}\;\mathrm{age}|}{n}$ was used to calculate the absolute error. In its simplest form, the absolute error ignores the sign (positive or negative) of the difference between the estimated and chronological ages [2, 6, 33]. Chi‐squared tests of independence were used to assess differences in the number of individuals classified correctly and incorrectly between methods and sexes; a *p*‐value of less than 0.05 was considered statistically significant.

For t‐tests, the dependent variable was the estimated age, and the independent variable was sex and laterality. To ascertain if each chronological age fell within the age ranges specified for each approach, the paired samples t‐test was utilized to compare the application of each age‐at‐death estimation method between males and females, and the p‐value was included to emphasize whether there was a significant difference in the application of each os coxa age‐at‐death estimation method in terms of accuracy between males and females. The three age‐at‐death estimation methods were evaluated for laterality using the paired samples *t*‐test, and the *p*‐value indicated whether there was a significant difference in the application of each age‐at‐death estimation method between the left and right os coxae. To determine whether these three approaches are consistent with their performance for estimating age‐at‐death, intraobserver error analysis was additionally incorporated into this study to get the intraobserver correlation coefficient. For each population, random selection of 30% of the sample was reassessed three times by the principal investigator. Ten days following the initial evaluation, the second re‐evaluation was conducted, and 15 days following the second re‐evaluation, the third re‐evaluation was conducted. The first evaluation of each approach was compared with the second and third evaluations for each population group using the Cronbach's alpha test. Then again, for each population, 15% of the sample was reevaluated by an independent observer (a senior researcher in the Discipline of Clinical Anatomy), where this evaluation was compared with the first evaluation by the principal investigator using the Cronbach's alpha test to get the interobserver error correlation coefficient, higher internal consistency as well as reliability are indicated by a higher Cronbach's alpha value, which is often closer to one.

## 3 RESULTS

### 3.1 The accuracy of the results through age distribution

**FIGURE 1:**
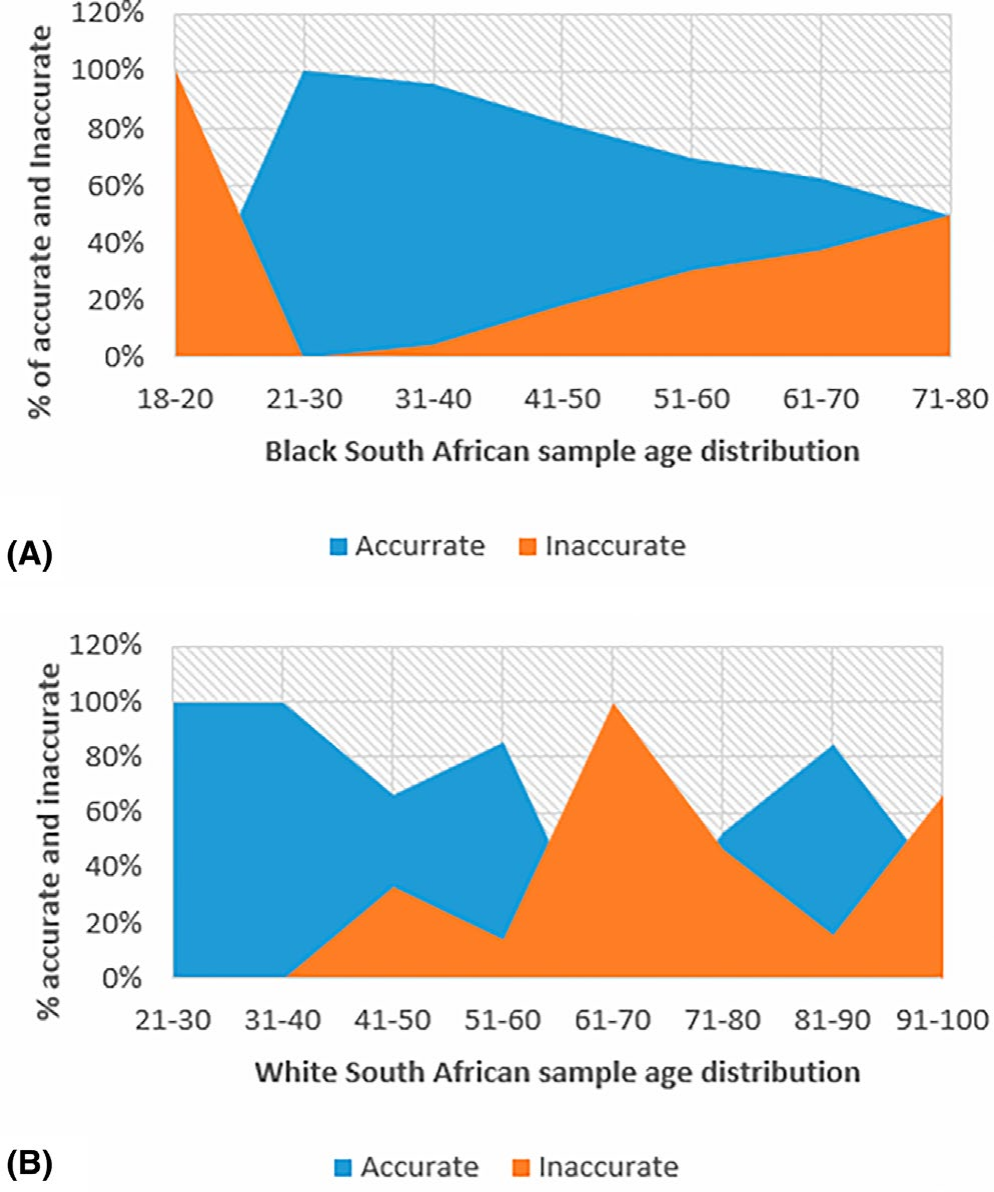
The area graph A and B representing the accuracy and inaccuracy of the pubic symphyseal surface method on age distribution in two South African population groups.

In the Black South African population, under the Suchey‐Brooks (pubic symphyseal surface) method, the age range of 18–20 years had the highest inaccuracy of 100%. The age range with the highest accuracy of 100% was 21–30 years and was followed by 31–40 years, with 95.7% (Figure 1A). In the White South African population, this method performed the best between the age range of 21 and 40 years, with an accuracy of 100% and performed poorly between the age range of 61 and 70, with an accuracy of 0% (Figure 1B).

**FIGURE 2:**
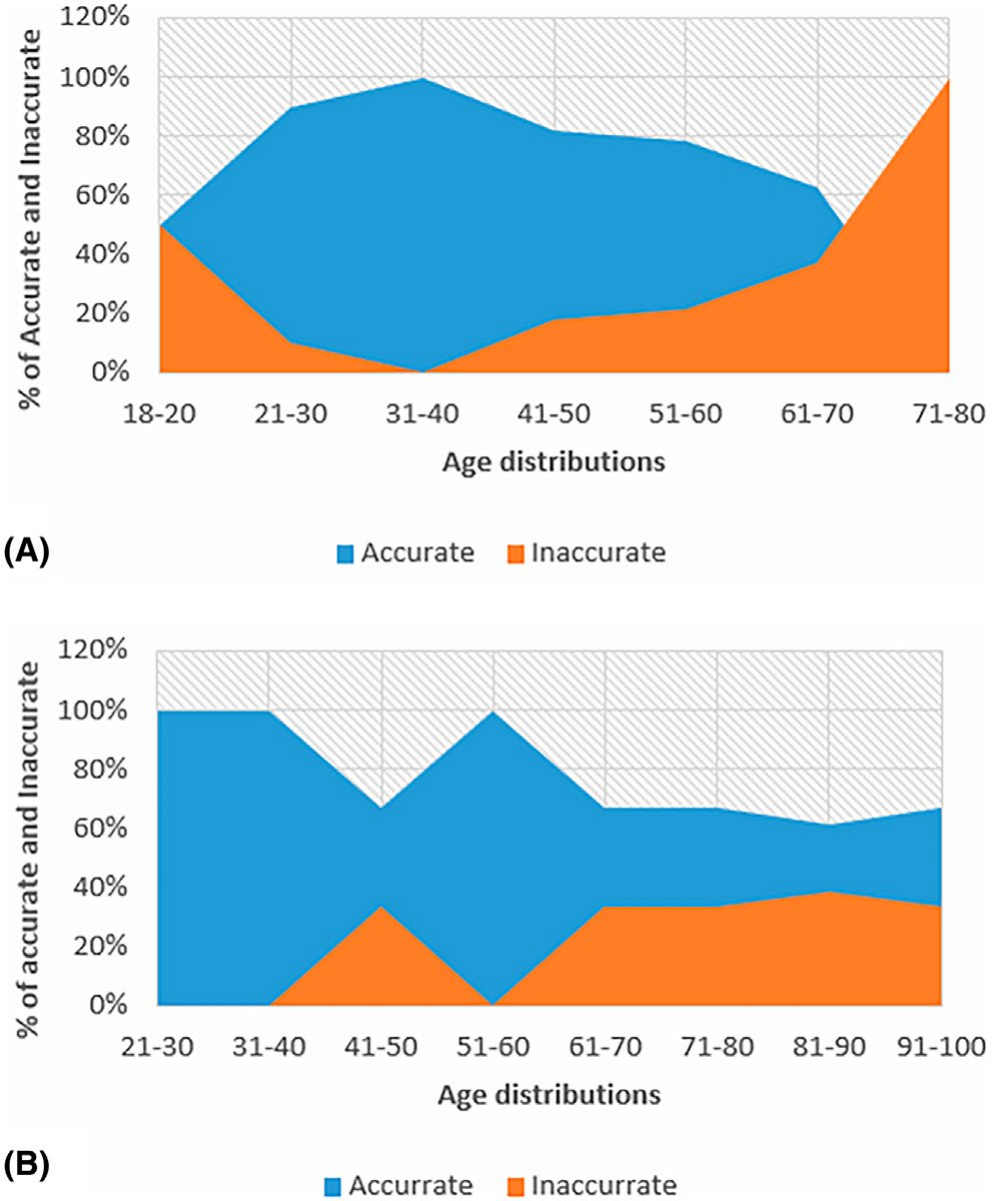
The area graph A and B representing the accuracy and inaccuracy of the auricular surface method on age distribution in two South African population groups.

In the Black South African population, under the Osborne (auricular surface) method, the age range of 71–80 years had the highest inaccuracy of 100%. The age range that scored 100% for accuracy was 31–40 years, with all os coxae estimated correctly, followed by a 90% accuracy of 21–30 years (Figure 2A). In the White South African population, the age range of 21–40 years and 51–60 years had the highest accuracy of 100% and 100%. The age range of 81–90 years had the highest inaccuracy of 38.46% (Figure 2B).

**FIGURE 3:**
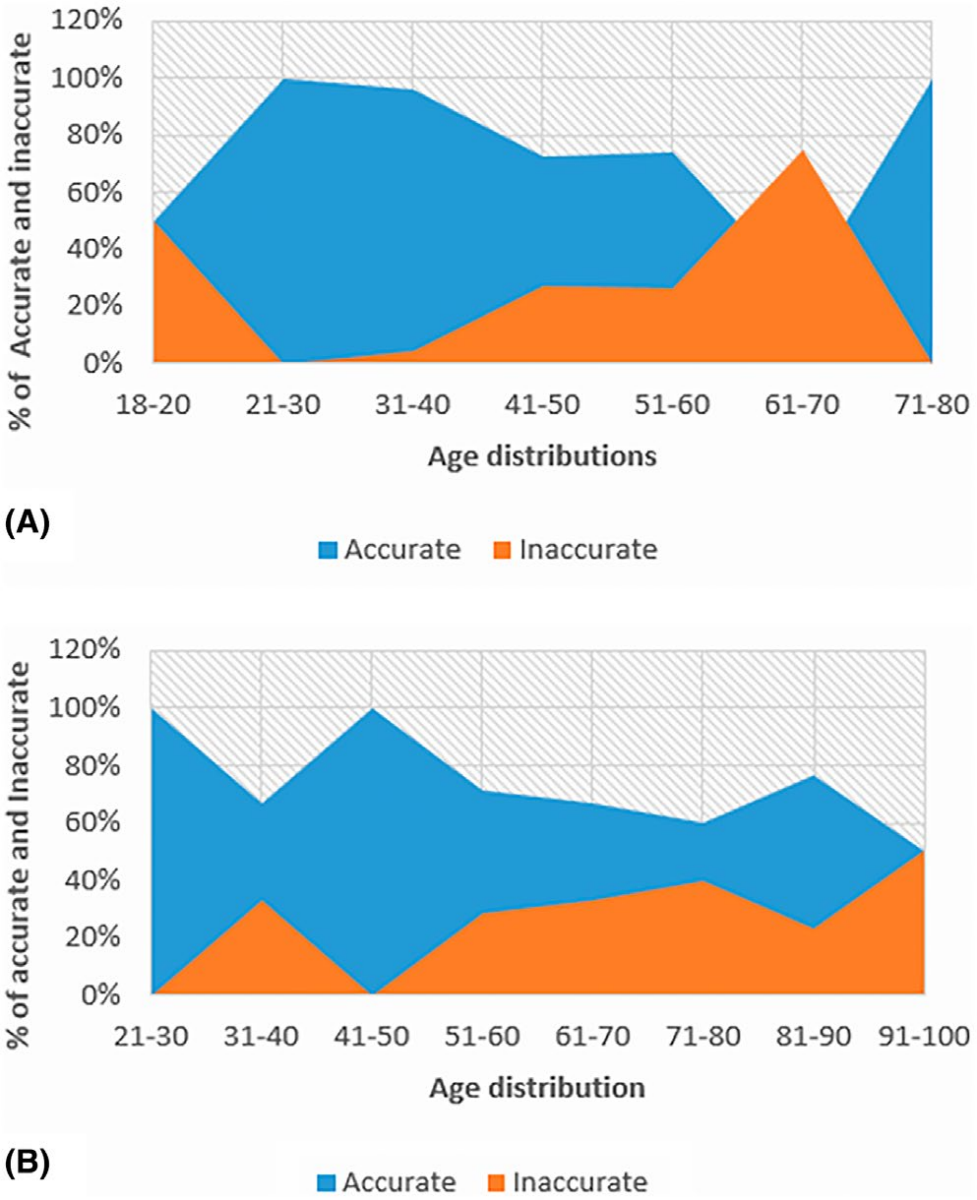
The area graph A and B representing the accuracy and inaccuracy of the acetabulum method on age distribution in two South African population groups.

In the Black South African population, the Calce (acetabulum) method performed better than the other two methods under the age distribution. The age range of 61–70 years had the highest inaccuracy of 75.0%. There were two age ranges with the highest accuracy of 100%, 21–30 years and 71–80 years. They were then followed by the age range of 31–40, with an accuracy of 95.7% (Figure 3A). In the White South African population, the acetabulum method showed good performance between the age ranges of 21–30 years and 41–50 years, with an accuracy of 100%. Then, the highest inaccuracy was seen in the age range of 91–100 years, 50% (Figure 3B).

### 3.2 Intra‐ and interobserver reproducibility

For intraobserver analysis, the first, second, and third evaluations of each age‐at‐death estimation method by the initial researcher were further analyzed through a Cronbach's alpha test to assess the correlation of the results upon reevaluation. The intra‐observer reproducibility for the acetabulum method showed good reliability when compared with the auricular surface and pubic symphyseal surface methods for both population groups, with the highest intraobserver correlation coefficient of 0.814 for the Black South African sample and 0.879 for the White South African sample. In the reevaluation of the pubic symphyseal surface method for the White South African population, poor reliability was found with the lowest intraobserver correlation of 0.266 (Table 4).

**TABLE 4:** Intraobserver variance.

	Intraobserver correlation coefficient	Confidence intervals 95%
Black South African sample		
Pubic symphyseal surface method	0.658	0.359–0.830
Auricular surface method	0.500	0.464–0.752
Acetabulum method	0.814	0.652–0.908
White South African sample		
Pubic symphyseal surface method	0.266	0.242–0.671
Auricular surface method	0.794	0.523–0.924
Acetabulum method	0.879	0.707–0.956

The independent observer results were compared with the first evaluation results from the initial researcher to find the interobserver correlation coefficient. For the pubic symphyseal surface and auricular surface, methods were found to be poor for both population groups. The acetabulum method outperformed the other two methods, with the highest interobserver correlation of 0.573 in the White South African population and 0.862 in the Black South African population (Table 5).

**TABLE 5:** Interobserver variance.

	Interobserver correlation coefficient	Confidence intervals 95%
Black South African sample		
Pubic symphyseal surface method	0.326	0.240–0.387
Auricular surface method	0.259	0.231–0.489
Acetabulum method	0.862	0.671–0.944
White South African sample		
Pubic symphyseal surface method	0.303	0.196–0.465
Auricular surface method	0.280	0.198–0.570
Acetabulum method	0.573	0.426–0.801

### 3.3 The analyses of the results through population groups and sex

The paired *t*‐test was used to determine the application of three age‐at‐death estimation methods between males and females and to determine if each chronological age fell within the age ranges given for each method. For the Black South African sample, the auricular surface method performed the best with the highest accuracy of 81.5%, followed by the pubic symphyseal surface method with 78.3% and lastly, the acetabulum method with 76.1% accuracy. When the results were analyzed in terms of sex for the Black South African sample, the auricular surface and pubic symphyseal surface methods performed better in females than in males, and the acetabulum method performed better in males than in females. The auricular surface method had accurately estimated age‐at‐death in females with 88.2% and in males with 77.6%; the pubic symphyseal surface method had 82.4% in females and 75.9% accuracy in males, and the acetabulum method had 73.5% in females and 77.6% accuracy in males. The p‐value demonstrated whether there is a significant difference in the application of each os coxa age‐at‐death estimation methods in terms of accuracy between male and female (Table 6). There was no statistical significance in the findings in all three methods (*p*‐values > 0.05).

**TABLE 6:** The performance of the three age‐at‐death estimation methods in the Black South African sample analyzed by sex.

Estimation method	Sex	Inaccurate		Accurate		Total	*p‐*value
		*n*	(%)	*n*	(%)	*n*	
Pubic symphyseal surface method	Male	14	24.1	44	75.9	58	
	Female	6	17.6	28	82.4	34	0.53
	Total	20	21.7	72	78.3	92	
Auricular surface method	Male	13	22.4	45	77.6	58	
	Female	4	11.8	30	88.2	34	0.2
	Total	17	18.5	75	81.5	92	
Acetabulum method	Male	13	22.4	45	77.6	58	
	Female	9	26.5	25	73.5	34	0.52
	Total	22	23.9	70	76.1	92	

The auricular surface method scored the highest accuracy of 72.5% when applied to the White South African sample, followed by the acetabulum method with 68.6%, while the pubic symphyseal surface method had the least accuracy of 64.7%. When the results were analyzed in terms of sex, the pubic symphyseal surface method performed better in females than in males, and the auricular surface and acetabulum methods performed better in males than in females. The auricular surface method had an accuracy of 70.4% in females and 75.0% in males, the acetabulum method scored 63.0% in females and 75.0% in males, and the pubic symphyseal surface method in females, 88.9% and in males, 37.5%. There was no significant difference in accuracy between males and females for the auricular surface and acetabulum methods (*p*‐values > 0.05). Statistical significance was noted between males and females for the pubic symphyseal surface method (*p* = 0.001) (Table 7).

**TABLE 7:** The performance of the three age‐at‐death estimation methods in the White South African sample analyzed by sex.

Estimation method	Sex	Inaccurate		Accurate		Total	*p‐*value
		*n*	(%)	*n*	(%)	*n*	
Pubic symphyseal surface method	Male	15	62.5	9	37.5	24	0.001
	Female	3	11.1	24	88.9	27	
	Total	18	35.3	33	64.7	51	
Auricular surface method	Male	6	25.0	18	75.0	24	0.71
	Female	8	29.6	19	70.4	27	
	Total	14	27.5	37	72.5	51	
Acetabulum method	Male	6	25.0	18	75.0	24	0.35
	Female	10	37.0	17	63.0	27	
	Total	16	31.4	35	68.6	51	

### 3.4 The analyses of the results through laterality and sex

The paired *t*‐test was used when assessing the application of the three age‐at‐death estimation methods in terms of laterality. For the Black South African sample, there were no cases where any of these methods performed better on the left side of the os coxa than on the right side; instead, these methods performed better when applied on the right side or were the same between the left and right sides in terms of accuracy. For both males and females, the pubic symphyseal surface method performed better on the right side with 79.5% accuracy and 77.1% on the left side, the auricular surface method showed 84.1% accuracy on the right side and 79.2% on the left side, and last, the acetabulum method had 81.8% on the right side and 70.8% left side (Table 8). When only laterality was observed, the right side outperformed the left side in all three methods for the Black South African sample. There was no statistical significance difference between left and right os coxae in all three methods (*p*‐values > 0.05) (Table 8).

**TABLE 8:** The performance of the three age‐at‐death estimation methods in terms of laterality and sex in the Black South African sample.

Estimation method	Sex	Left side					*p*‐value	Right side				
		Inaccurate		Accurate		Total		Inaccurate		Accurate		Total
		*n*	(%)	*n*	(%)			*n*	(%)	*n*	(%)	*n*
Pubic symphyseal surface method	Male	8	25.8	23	74.2	31	0.77	6	22.2	21	77.8	27
	Female	3	17.6	14	82.4	17		3	17.6	14	82.4	17
	Total	11	22.9	37	77.1	48		9	20.5	35	79.5	44
Auricular surface method	Male	8	25.8	23	74.2	31	0.54	5	18.5	22	81.5	27
	Female	2	11.8	15	88.2	17		2	11.8	15	88.2	17
	Total	10	20.8	38	79.2	48		7	15.9	37	84.1	44
Acetabulum method	Male	9	29.0	22	71.0	31	0.31	4	14.8	23	85.2	27
	Female	5	29.4	12	70.6	17		4	23.5	13	76.5	17
	Total	13	29.2	35	70.8	48		8	18.2	36	81.8	44

The accuracy in terms of laterality in the White South African sample demonstrated that the auricular surface and acetabulum methods performed better on the right os coxa than on the left, and vice versa for the pubic symphyseal surface method. The pubic symphyseal surface method had an accuracy of 61.9% on the right and 66.7% on the left os coxa, the auricular surface method had 76.2% on the right and 70.0% on the left os coxa, and the acetabulum method had 76.2% on the right and 63.3% accuracy on the left os coxa. There was no statistical significant difference between left and right os coxae in all three methods (*p*‐values > 0.05) (Table 9).

**TABLE 9:** The performance of the three age‐at‐death estimation methods in terms of laterality and sex in the White South African sample.

Estimation method	Sex	Left side					*p* value	Right side				
		Inaccurate		Accurate		Total		Inaccurate		Accurate		Total
		*n*	(%)	*n*	(%)	*n*		*n*	%	*n*	%	*n*
Pubic symphyseal surface method	Male	9	69.2	4	30.8	13		6	54.5	5	45.5	11
	Female	1	5.9	16	94.1	17		2	20.0	8	80.0	10
	Total	10	33.3	20	66.7	30	0.77	8	38.1	13	61.9	21
Auricular surface method	Male	4	30.8	9	69.2	13		2	18.2	9	81.8	11
	Female	5	29.4	12	70.6	17		3	30.0	7	70.0	10
	Total	9	30.0	21	70.0	30	0.54	5	23.8	16	76.2	21
Acetabulum method	Male	2	15.4	11	84.6	13		4	36.4	7	63.6	11
	Female	9	52.9	8	47.1	17		1	10.0	9	90.0	10
	Total	11	36.7	19	63.3	30	0.75	5	23.8	16	76.2	21

### 3.5 The summary of the results on accuracy, bias, and absolute error

The mean absolute error (MAE) and Wilcoxon signed rank test were conducted. Regardless of whether age is overestimated or underestimated, the term “absolute error” describes the mean average error in years, and “bias” is the average error in years that considers the direction of the deviation. The pubic symphyseal surface method underestimated age by 1.36 years and had an absolute error of 13.70 years; the acetabulum method overestimated age by 1.90 years and had an absolute error of 13.15 years; the auricular surface method overestimated age by 5.98 years and had an absolute error of 21.05 years for the Black South African sample (Table 10).

**TABLE 10:** The summary age of accuracy, absolute error, and bias of each age‐at‐death estimation method in the Black South African sample.

Estimation method	Sex	*n*	Mean	SD	Accuracy (%)	Inaccuracy (%)	Bias (years)	Absolute error (±years)
True age	Males	58	46.57	15.31	–	–	–	–
	Females	34	44.29	11.45	–	–	–	–
	Total	92	45.73	13.99	–	–	–	–
Pubic symphyseal surface method	Males	58	48.41	15.07	75.9	24.1	−1.84	11.97
	Females	34	44.82	16.66	82.3	17.6	−0.53	15.17
	Total	92	47.09	15.68	78.3	21.7	−1.36	13.17
Auricular surface method	Males	58	39.52	14.65	77.6	22.4	7.05	22.18
	Females	34	40.15	12.62	88.2	11.8	4.15	19.14
	Total	92	39.75	13.86	81.5	18.5	5.98	21.05
Acetabulum method	Males	58	45.22	16.12	77.6	22.4	1.34	12.32
	Females	34	41.44	17.48	73.5	26.5	2.85	14.61
	Total	92	43.83	16.64	76.1	23.9	1.90	13.15

All three methods in the White South African sample underestimated age, with 11 years for the pubic symphyseal surface method and an absolute error of 14.51 years. The auricular surface approach had an underestimation value of 11.39 years and an absolute error of 16.65 years, and the acetabulum method had a value of 9.06 years and an absolute error of 12.63 years (Table 11).

**TABLE 11:** The summary age of accuracy, inaccuracy, and bias of each age‐at‐death estimation method in the White South African population.

Estimation method	Sex	*n*	Mean	SD	Accuracy (%)	Inaccuracy (%)	Bias (years)	Absolute error (±years)
True age	Males	24	71.21	19.65	–	–	–	–
	Females	27	74.52	16.18	–	–	–	–
	Total	51	72.96	17.79	–	–	–	–
Pubic symphyseal surface method	Males	24	55.00	14.40	37.5	62.5	−16.21	18.63
	Females	27	67.59	14.40	88.9	11.1	−6.93	10.85
	Total	51	61.67	15.61	64.7	35.3	−11.00	14.51
Auricular surface method	Males	24	56.71	20.09	75.0	25.0	−14.50	18.33
	Females	27	65.89	14.01	70.4	29.6	−8.63	15.15
	Total	51	61.57	17.58	72.4	27.5	−11.39	16.65
Acetabulum method	Males	24	65.96	17.41	75.0	25.0	−5.25	11.17
	Females	27	62.07	16.73	63.0	37.0	−12.44	13.93
	Total	51	63.90	17.00	68.6	31.4	−9.06	12.63

## 4 DISCUSSION

Finding reliable methods to estimate age‐at‐death from adult skeletal remains is challenging because the accuracy of age estimation can be unreliable when aging methods created for one population are used on another [6, 21]. When the reference and test populations are separated by more time and distance, the accuracy of age‐at‐death estimates decreases due to factors such as the individual's origin, nutrition, and physical activities, thus resulting in skeletal variability in different populations [8, 34]. Therefore, testing different age‐at‐death estimation methods on different population groups is significant; for instance, the Suchey‐Brooks [13] method, which is still popular, was created with a White population in North America and may not be suitable for other population groups. To use this method in a different population group, it requires testing for accuracy and repeatability before the experts in forensic science, anthropologists, and police departments entrust it to yield accurate identification results [6].

The purpose of this study was to compare the accuracy of three (viz., pubic symphyseal surface, auricular surface, and acetabulum) age‐at‐death estimation methods when applied to the Black South African and White South African population groups in the KZN province, South Africa.

### 4.1 The overall performance accuracy of the three age‐at‐death estimation methods

#### 4.1.1 Accuracy on population groups

Based on the current study findings for the Black South African sample, the auricular surface method yielded the highest accuracy, followed by the pubic symphyseal surface method, and the acetabulum method had the least accuracy. The rankings in terms of performance accuracy of these methods align with the results of a comparative study by Miranker [6] in the United States (Table 12). The three methods performed poorly when applied to the White South African sample—they scored low accuracy compared to when they were applied to the Black South African sample. On the White South African sample, the auricular surface method had the highest accuracy, followed by the acetabulum method, and the pubic symphyseal surface method had the least accuracy [6] (Table 12).

**TABLE 12:** The results of previous studies (accuracy, bias, and absolute error).

Author(s)	Population	Sex	Sample size	Estimation methods	Accuracy (%)	Bias (years)	Absolute error (years)
Hens et al. [38]	Italy	Males and females	404	Pubic symphyseal surface Auricular surface method	– –	−18.90 −15.60	27.40 24.20
Rissech et al. [2]	Spain	Males and females	217	Pubic symphyseal surface	71	−2.45	8.21
Miranker [6]	United States	Males and females	206 210 212	Auricular surface Pubic symphyseal surface Acetabulum	86.89 64.76 59.43	−13.86 −18.77 −5.46	15.61 19.27 13.18
Jones et al. [37]	South Africa	Males and females	394	Auricular surface, Pubic symphyseal surface	– –	6.20 −0.50	13.80 10.60
Current study	South Africa	Males and females	Black South Africans 92 White South Africans 51	Auricular surface Pubic symphyseal surface Acetabulum Auricular surface Pubic symphyseal surface Acetabulum	81.50 78.30 76.10 72.40 64.70 68.60	5.98 −1.36 1.90 −11.39 −11.00 −9.06	21.06 13.17 13.15 16.65 14.51 12.63

Even though it was expected that the pubic symphyseal surface method would perform better than the other two methods, as it is widely used in the forensic community [6], previous literature introduced doubt about the accuracy of this method [6, 35]. Godde and Hens [24] compared the accuracy of transition analysis combined with a Bayesian approach to the standard Suchey‐Brooks age ranges on the Italian population from the island of Sardinia, owing to the challenge of determining a suitable informative prior for bioarchaeological specimens. The results indicated that the Bayesian method fared better than the Suchey‐Brooks method alone, and it was suggested that bioarchaeologists must use an informative prior to age historic and archaeological populations using a Bayesian technique to increase accuracy [24]. The findings from the current study align with the abovementioned literature, as this method was second in terms of accuracy when applied to the Black South African sample and third when applied to the White South African sample. Additionally, it was expected for the pubic symphyseal surface method to perform poorly when applied to the White South African sample in this current study, as this method is known to be more accurate in younger individuals 18–40 years [15], and the White South African sample in the current study had a high proportion of individuals who were above 40 years of age. Martrille et al. [33] also documented that the Suchey‐Brooks method was the most accurate for individuals aged 25–40 when the sample was divided into age groups. This might have favored the Osborne auricular surface method to score the highest accuracy, due to the age ranges developed for this method being very broad (as phase IV ranges between 20–75 years), thus making it difficult for incorrectly estimating age, as this age range almost covers young, middle, and old adult groups.

The auricular surface is regarded as a good age indicator because its morphology is independent of population affinity or sex; therefore, an estimate of an individual's age‐at‐death can be made without knowledge of their population affinity or sex [16, 36]. Additionally, because the auricular surface is resistant to taphonomic processes, it can be useful when fragmentary remnants are present [14, 15], and this might be the reason why the auricular surface method outperformed the pubic symphyseal surface and acetabulum methods when examined on both the Black South African and White South African samples in terms of accuracy in the current study. When the results were broken down into age groups, all three os coxa age‐at‐death estimation methods were performing better in terms of accuracy from the age of 21–40 years, as all their accuracies were above 60% in both population groups. Previous studies have documented that accuracy decreases with increasing age. The Suchey‐Brooks pubic symphyseal surface method was reported to be more accurate in younger individuals 18–40 years [15]. Miranker [6] also noted that the middle age range of 41–80 years of age was less accurately represented by acetabulum methods, which has never been documented before. It became clear that accuracy declined in all methods after 60 years, both for the entire sample and when broken down into age groups [6].

#### 4.1.2 Repeatability of the results

The acetabulum method had higher repeatability values than the auricular surface and pubic symphyseal surface methods in both population groups, indicating that examining the acetabulum method is easier compared to the auricular surface and pubic symphyseal surface methods. These results were also supported by Jones [37], who documented that it is frequently challenging to differentiate between certain features on the auricular surface and the pubic symphyseal surface. Hens [38] examined the pubic symphyseal surface and auricular surface methods on one sample and found that the auricular surface method performed better than the pubic symphyseal surface method in terms of bias and absolute error (Table 12). It was further documented that morphological changes observed with increasing age for the auricular surface method are more complex compared to those of the pubic symphyseal surface, and many inexperienced observers may find it difficult to give accurate results [38]. The current study results support the above author as the intraobserver repeatability values were slightly higher than the interobserver repeatability values. Botha [39] also documented that since most adult age estimation techniques are qualitative and subject to interpretation, scoring consistency, interobserver repeatability, and reliability are generally significant issues. All different traits change gradually, and it is not always obvious or precise when one stage ends and another begins. For instance, in sternal rib end and pubic symphysis analysis, detailed descriptions, drawings, and casts have been employed to aid the observer; nonetheless, even among experienced observers, there appears to be significant heterogeneity in how a single case is scored [39].

#### 4.1.3 Laterality

Furthermore, laterality was also assessed in the current study because there is a paucity of studies on age‐at‐death estimation methods when using the os coxae. Most studies have followed previous studies by assessing the left os coxa and inspecting the right os coxa only when the region of interest on the left is too damaged or otherwise unavailable [15]. The left and right sides' pelvic aging sites do not seem to differ significantly according to different authors [4, 6, 15, 17, 21, 33, 39]. The current study supports the abovementioned authors as no statistical significance difference was noted in the three aging methods on the left or right sides of the os coxa for both the Black South African and White South African samples.

#### 4.1.4 Sex

Even though sex is deemed insignificant between the auricular surface and acetabulum methods, it is known to be a very important factor in the pubic symphyseal surface method, as it has different age ranges for males and females [6, 13, 15, 30]. The results from the current study support the abovementioned authors as there was no statistical significance in the performance accuracy between sexes for both Black South African and White South African samples. Numerous investigations have examined the skeleton to identify variations between males and females [40, 41, 42], and sexual dimorphism is known to be population‐specific due to human reproduction and other factors [42]. The current study supports the previous literature, as the p‐value is less than 0.05 for the pubic symphyseal surface method on the White South African sample, indicating that the performance of this method differs significantly between males and females. Factors such as physical activity (i.e., exercise, leisure, manufacturing, entertainment, recreation, and war) and hormonal changes are some of the main causes of the morphological changes observed on the pubic symphysis [43]. Pubic bone resorption is caused by elevated estrogen levels, such as those experienced during pregnancy and menstrual cycles [44].

#### 4.1.5 Bias and absolute error

“Absolute error” refers to the mean average mistake in years, regardless of whether age is overestimated or underestimated, whereas “bias” refers to the average error in years that takes the deviation's direction into account [2, 33]. Although the auricular surface method showed good performance accuracy, it also scored the highest absolute error and bias, making it the least trusted method. The method with the lowest absolute error and bias in both the Black South African and White South African samples was the acetabulum method. This makes the acetabulum the most trusted indicator, as Miranker [6] reported the lowest absolute error and bias (±13.18 and −5.46 years) in the acetabulum method compared to the auricular surface method (±15.61 and −13.86 years) and pubic symphyseal surface method with the highest absolute error and bias (±19.27 and −18.77 years) where overall age for all three methods was underestimated. Jones [37] also reported high absolute error and bias (±13.80 and 6.20 years) for the auricular surface compared to the pubic symphyseal surface (±10.60 and −0.50 years) (Table 12).

The measures of bias and absolute error were added to show how far the mean point age estimate deviates from the known age, as accuracy may be misleadingly high. The current study findings may be due to the age ranges created for the auricular surface and pubic symphyseal surface methods being very broad, corresponding to six age phases. For instance, an estimated age for the auricular surface and pubic symphyseal surface methods may fit into more than one age range. While the acetabulum method has only three morphological phases corresponding to three age ranges, this restricts an estimated age from fitting into more than one age range. For the pubic symphyseal surface, the Bayesian age ranges for various coverages have been provided by Godde and Hens [45] in the study that examined the Bayesian approach to the pubic symphyseal surface method. The Bayesian methodology, along with transition analysis, as anticipated, improved age estimations, particularly for females, compared to raw data for the Suchey‐Brooks technique. It was also documented that the Bayesian approach demonstrated advantages that include control for bias reporting, age mimicry, and actual accuracy levels attained [45].

Rissech [4] emphasized that damaged acetabula should not be utilized. The sample size should be large and from the same geographical region; these conditions must be met to get reliable results from the acetabulum method. Hens [38] documented the high bias and absolute error with increasing age for pubic symphyseal surface and auricular surface methods, and overall age for both methods was underestimated. Saunders et al. [46], while testing four age‐at‐death estimation methods, also documented the increasing bias and absolute error with increasing age. Rissech [2] also underestimated the overall age while examining the pubic symphyseal surface on the Spanish sample (Table 12). In the current study, all three age‐at‐death estimation methods were found to underestimate age in the White South African sample, and only the pubic symphyseal surface method was found to underestimate age in the Black South African sample.

## 5 CONCLUSION

The overall findings of the current study suggested that these methods are still lacking, as the results on both population groups demonstrated that the auricular surface method was better in terms of accuracy than the other two methods. However, this method also scored the highest bias and absolute error. Hence, the result from this study preferred the acetabulum method to be used in Black South African and White South African population groups in KZN, South Africa over the pubic symphyseal surface and auricular surface methods, as this method showed to maintain lower bias and absolute error on both population groups. Furthermore, the acetabulum method also tended to be the most reliable in terms of consistency for repeatability of the results when evaluated in both population groups. Sex was insignificant when the auricular surface and acetabulum methods were employed; however, sex may be a significant factor when the pubic symphyseal surface method is used, as its accuracy was found to be insignificant for the Black South African sample but significant for the White South African sample. These results show the importance of developing sample references with traits for each os coxa age‐at‐death estimation method that can cater to different South African population groups to increase the accuracy, and age ranges and phases be reduced for the auricular surface and pubic symphyseal surface methods to decrease their bias and absolute error. Also, employing the Bayesian technique to all three os coxa methods is suggested for future studies in different populations.

## FUNDING INFORMATION

National Research Foundation (NRF), Grantee: Sthembiso Mkhonza; Grant Number: PMDS22052313937. Hereby, the NRF's financial support for this research is recognized. The authors' opinions and conclusions are their own, and the NRF should not always be credited with them.

## CONFLICT OF INTEREST STATEMENT

The authors have no conflict of interest to declare.

## ETHICS STATEMENT

This study was approved by the Biomedical Research Ethics Committee (BREC) of the University of KwaZulu‐Natal (UKZN). The ethical clearance number is BREC/00006368/2023.

## Data Availability

The data that support the findings of this study are available from the corresponding author upon reasonable request.
